# Digital Health Value Realization Through Active Change Efforts

**DOI:** 10.3389/fpubh.2021.741424

**Published:** 2021-10-12

**Authors:** Rashaad Bhyat, Simon Hagens, Katie Bryski, Jocelyn Fausto Kohlmaier

**Affiliations:** Canada Health Infoway, Toronto, ON, Canada

**Keywords:** digital health, value realization, change efforts, electronic health record, virtual care, digital tool adoption, benefits - case study

## Abstract

Digital health has massive potential in health care but has been slow to evolve in comparison to other information-intensive industries, which have more readily taken advantage of new technology. One of the key barriers has been the complex relationship between the perceived return on investment for the investor and the resulting value to patients and caregivers. Those actors who pay for technologies do not always see an appreciable return for themselves, while those actors who must apply the technology to generate value are not always incentivized to do so. This misalignment across health system payers and administrators, clinicians and patients must be better understood and addressed to help accelerate digital health. This paper will examine this challenge through the clinician experience, using empirical case examples from Canada to illustrate opportunities for change. While many factors may influence digital health adoption, this paper specifically aims to explore the shifts in the balance of the perceived value of implementing digital health tools, vs. the efforts required to adopt them. It will explore two contrasting case examples: clinical adoption of EMRs in Canada from 2009 to 2015, and clinical adoption of virtual care technologies during the COVID-19 pandemic from 2020 to 2021. In 2006, Canada lagged peer countries significantly in the adoption of electronic medical records (EMR) in community-based care. Financial support and cooperation of multiple levels of government and clinical stakeholders were required to address the misaligned incentives, which led to significant uptake by care providers. The rapid adoption of virtual care in Canada in response to the pandemic provides another relevant example of the importance of alignment among the factors of clinical workflows, clinical appropriateness, technology integration and payment models. Experts have highlighted the need for standardization, regulation, and clear policy to ensure sustainable, high quality virtual care that complements in-person care. In both cases, the costs and effort of adopting new technologies outweighed direct clinician value, requiring change initiatives to catalyze progress. This imbalance could be unique to these examples in Canada, and may not be globally generalizable to the adoption of all digital health tools. However, how change efforts can be tailored to adjust to a rapidly evolving health care workforce, spanning diverse jurisdictions and stakeholder groups will be critical to the sustainability of virtual care adoption. Furthermore, what key elements must be considered to guide change initiatives for successful implementation, designed to influence change while adding value for patients, clinicians and Canada's health care systems? Using insights from successful change initiatives past and present, this paper aims to answer these questions to enable a smoother transition to digital health innovations of the future.

## Introduction

“*One essential characteristic of modern life is that we all depend on systems—on assemblages of people or technologies or both—and among our most profound difficulties is making them work*.”

- Dr. Atul Gawande, The Checklist Manifesto: How to Get Things Right

Digital health, particularly virtual care, holds significant promise for modernizing health care delivery in Canada. However, the value proposition derived from implementing digital health tools is complex in the Canadian health care setting.

Within the Canadian context, those players that are responsible for funding the adoption and use of digital health technologies (such as government bodies) do not always see immediate, appreciable value for themselves, while the actors (e.g., clinicians) who must adopt the technology in order to generate that value are not always incentivized to do so. The barriers to digital health adoption can therefore appear greater than the benefits resulting from more widespread use.

Digital health tools and initiatives can potentially add value to the health system by helping to achieve the goals of health care's Quadruple Aim (1): improving the health of the population, improving the patient experience, reducing costs and improving the health care provider experience.

Despite this potential for a positive impact, Canada has historically lagged peer nations with regard to integrating digital tools and services into its health system, as noted in Commonwealth Fund surveys (2). The costs of adopting new technologies (monetary costs, as well as time and effort) must not outweigh direct value to clinicians and must have clear benefits to patients. In cases where costs may be perceived as outweighing benefits, change initiatives are required to catalyze progress and “balance the scales.” To reach success and maturation, these change initiatives must present a compelling value proposition to the technology's adopters.

Traditionally, articulating this value proposition to clinicians has proven challenging, as demonstrated through analyzing Canada's experience implementing electronic medical records (EMRs). As a result of these challenges in driving adoption, digitization in Canadian health care has been slower than in other industries, such as banking.

However, the onset of the COVID-19 pandemic in March 2020 presented an emergent, highly compelling value proposition to clinicians. The implementation of physical distancing measures to slow the transmission of the virus created an urgent need to reduce in-person contact and to keep patients out of crowded waiting rooms. Fewer in-person interactions could lower patient and provider risk of exposure to the virus. Health systems and clinicians thus faced a sudden urgency and necessity to integrate virtual care technologies into care delivery.

Newly available temporary provincial and territorial billing codes no longer disadvantaged clinicians for providing care virtually (3). Where appropriate, patients could access care from their physician remotely, keeping all parties safe from the inherent risks of physical contact. While multiple clinical organizations initiated change efforts in the form of virtual care best practice guides and implementation toolkits, these strategies were short-term in nature. Indeed, they were responses to an emergency situation.

While many factors may influence digital health adoption (4), this paper specifically aims to explore the shifts in the balance of the perceived value of implementing digital health tools, vs. the effort required to adopt them. It explores two contrasting empirical case examples: clinical adoption of EMRs in Canada from 2009 to 2015, and clinical adoption of virtual care technologies during the COVID-19 pandemic from 2020 to 2021.

International analysis of clinical engagement in digital health conducted by the Global Digital Health Partnership has found that while contexts and technology adoption differ around the globe, clinician change challenges and requirements for creating value are common (5). Nonetheless, it is important to note that while in these two cases, the costs and effort of adopting new technologies initially outweighed direct clinician value, this imbalance could be unique to the context of the Canadian health care system and may not be globally generalizable to the adoption of all digital health tools.

## Who Benefits from Digital Health?

There is a longstanding business case for investments in digital health. Cited benefits range from the basic efficiencies of productivity (task automation, for example), to enhancements in patient safety (6), to opportunities to improve the quality of patient care and the health of populations.

In Canada, the uptake of digital health has been gradual and unevenly distributed across health care settings and among health professionals, including nurses, physicians and pharmacists.

**Figure 1:**
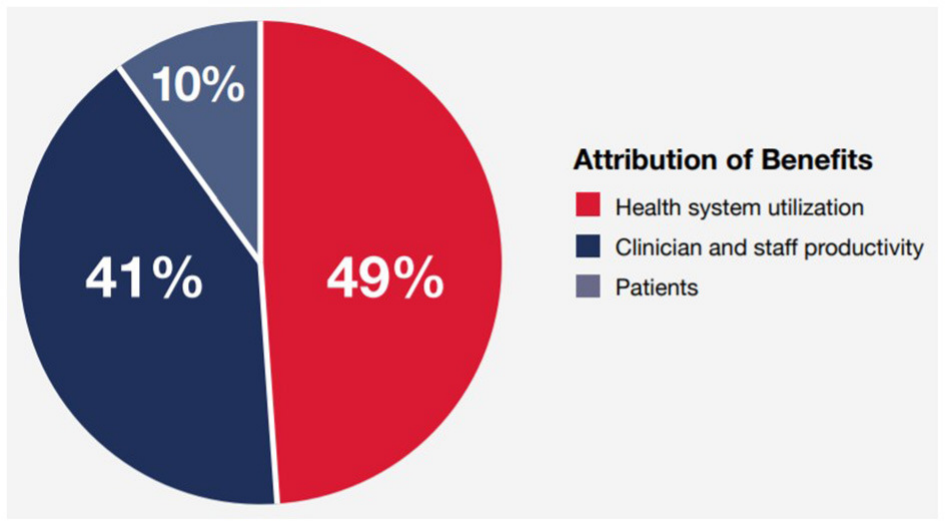
Portion of Digital Health benefits flowing to patients, clinicians and health systems in 2019. Infoway Annual Report 2018–2019. Sourced from Digital Health outcomes models developed between 2008 and 2021 to estimate impacts in Canada (8).

Canada Health Infoway (Infoway) developed a methodology for estimating benefits from a defined set of digital health solutions (7). This analysis illustrates the distribution of those benefits between patients and caregivers, clinicians and their staff, and health systems. As Figure 1 shows, results collected prior to the COVID-19 pandemic demonstrate that health systems recoup half the value, followed by clinicians with another substantial share (8). Patients received only 10% of the estimated value. However, changes in health care delivery throughout the pandemic, notably the shift to virtual care and heightened use of digital health tools, are seeing patients receive an increasing share of that value.

As the Canadian experience demonstrates, technological infrastructure alone is insufficient to generate the momentum for widespread clinical adoption of digital health technologies—so long as the value to clinicians is not evident. Digitization in itself does not necessarily lead to functioning, clinician-friendly digital systems.

As a result, sluggish adoption of digital health by clinicians has a cascading effect on potential benefits to patients. The benefits of technological efficiencies cannot be realized if those technologies are not in use, or if their use is not optimized for enhancing patient care and the patient experience.

In the early 2000s, Canadian provinces and territories implemented the foundational elements of digital health, including the gradual introduction of provincial/territorial electronic health record (EHR) systems, hospital information systems (HIS) and community-based electronic medical records (EMRs) in physicians' offices.

**Figure 2:**
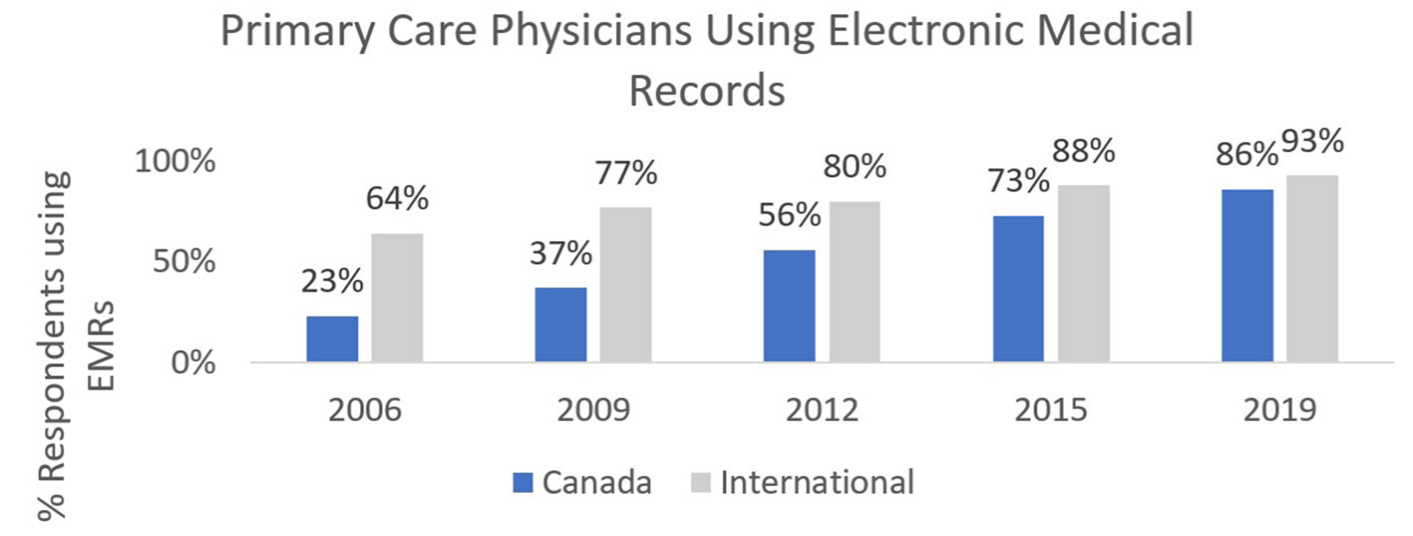
Canadian and International Primary Care Physician adoption of Electronic Records from 2006–2019. Sourced from the Commonwealth Fund International Survey of Primary Care Physicians, which surveyed 500 or more primary care physicians in each of 11 countries every 3–4 years from 2006–2019 (2).

As Figure 2 demonstrates, a combination of federal, provincial and clinician-based funding helped to increase EMR adoption in primary care nationally, with some variation between jurisdictions. These early digitization efforts resulted in many siloed clinical information systems across the country: at the jurisdictional, health authority, hospital and individual clinician practice levels. Systems could not and did not easily share information with each other, limiting their early utility and value proposition. Additionally, clinician challenges with multiple logins for disconnected systems led to frustration, possibly contributed to burnout (9) and further limited the value proposition of these digital tools for clinicians of all disciplines.

**Figure 3:**
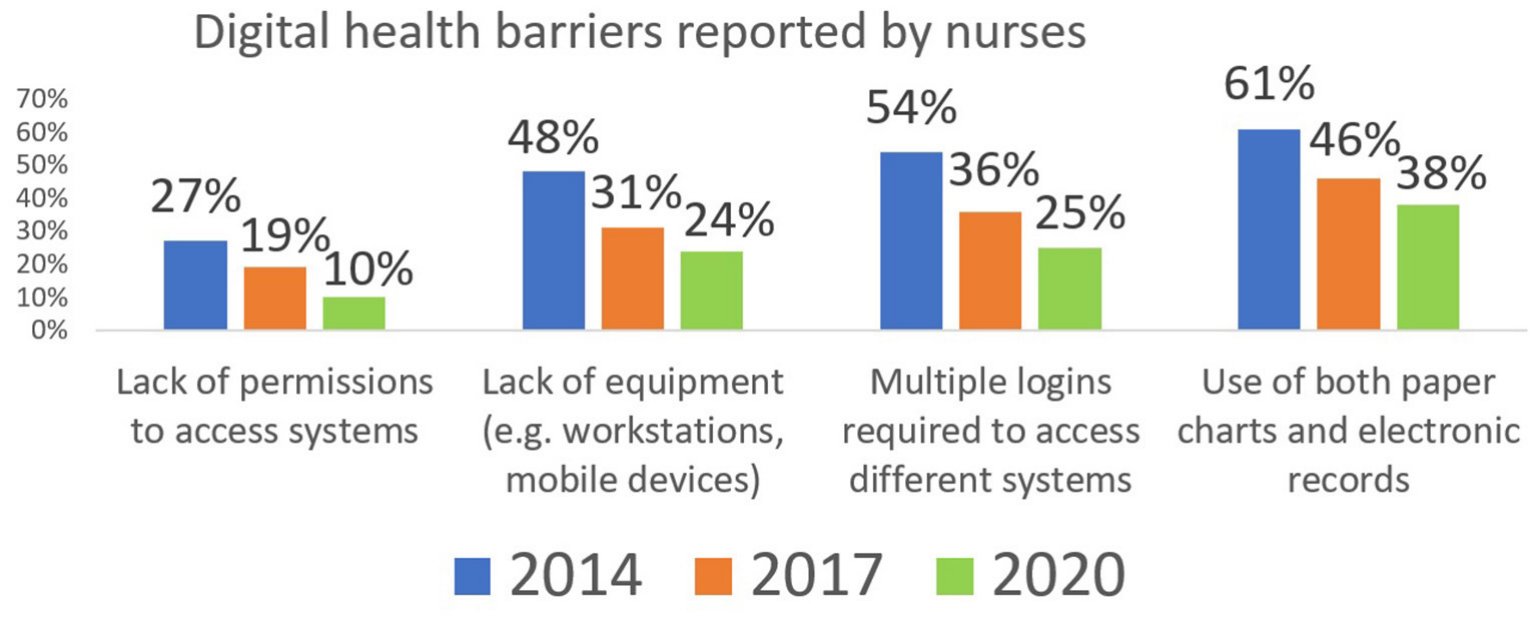
Portion of Canadian Nurses Reporting Digital Health barriers. Sourced from the national surveys of Canadian Nurses, with responses from over 1,500 nurses in each of 2014, 2017 and 2020 (10).

As noted in Figure 3, Canadian nurses reported challenges in multiple domains relating to digital health. While efforts to increase interoperability have led to improvements in recent years, many of these challenges persist. As a result, nurses and many other clinicians have had to adapt their practices to digital systems which were not designed for their unique clinical workflows. For these clinicians, the added physical and mental effort required to adapt to these digital systems might outweigh any perceived value for themselves and for their patients.

The lack of a compelling value proposition is compounded by the remuneration models for large groups of clinicians, specifically physicians. Despite efforts to reform payment models in Canada's health system, most physicians (73%) in both primary and specialty care in Canada operate under some form of a fee-for-service (FFS) model (11). Essentially, they operate as individual small-to-medium-size businesses and are responsible for any investments into their own technological infrastructure.

**Figure 4:**
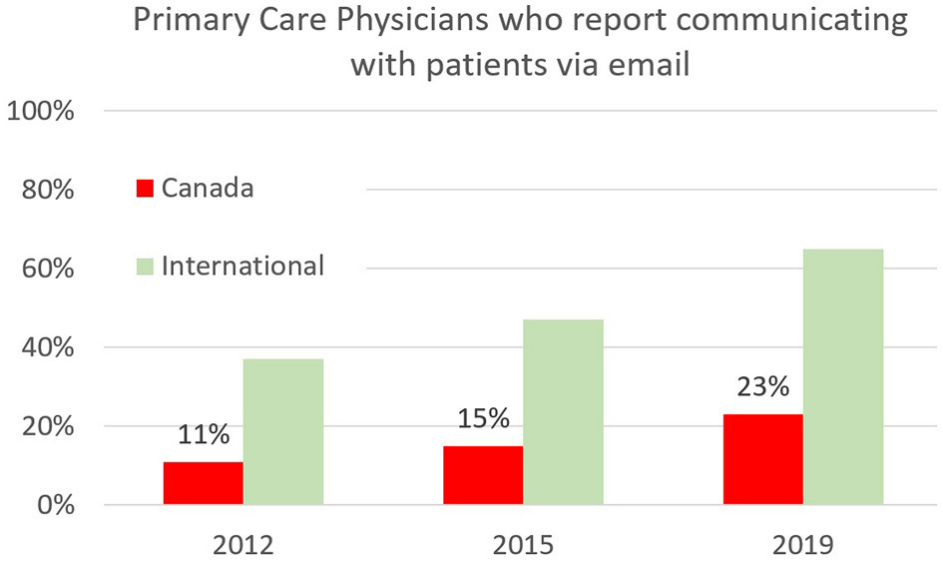
Primary Care Physicians in Canada and internationally who report offering patients the option to communicate via email from 2012–2019. Sourced from the Commonwealth Fund International Survey of Primary Care Physicians, which surveyed 500 or more primary care physicians in each of 11 countries every 3–4 years from 2006–2019 (2).

Prior to the pandemic, Canadians often wondered why it was challenging to email their physicians (12). As seen in Figure 4, only 23% of physicians reported communicating with patients by email in 2019. The 2018 Canadian Physician Survey (CPS) provided insight into the reasons behind this modest implementation of a seemingly basic form of communication. Physician remuneration structures had not kept pace with technological changes, nor with society's expectations of modern communication.

In Canada's single payer health system, physicians are remunerated by provincial or territorial ministries of health and cannot unilaterally adjust the cost of their services to offset technological infrastructure investments. For example, a family physician cannot charge the government more for seeing a patient in her office to offset the upfront cost of implementing a new EMR, nor can she charge for communications via a patient-facing secure messaging tool if a government-endorsed fee code does not exist for this type of communication. Without compelling evidence that these digital tools could enhance patient care, the impetus to change was limited.

**Figure 5:**
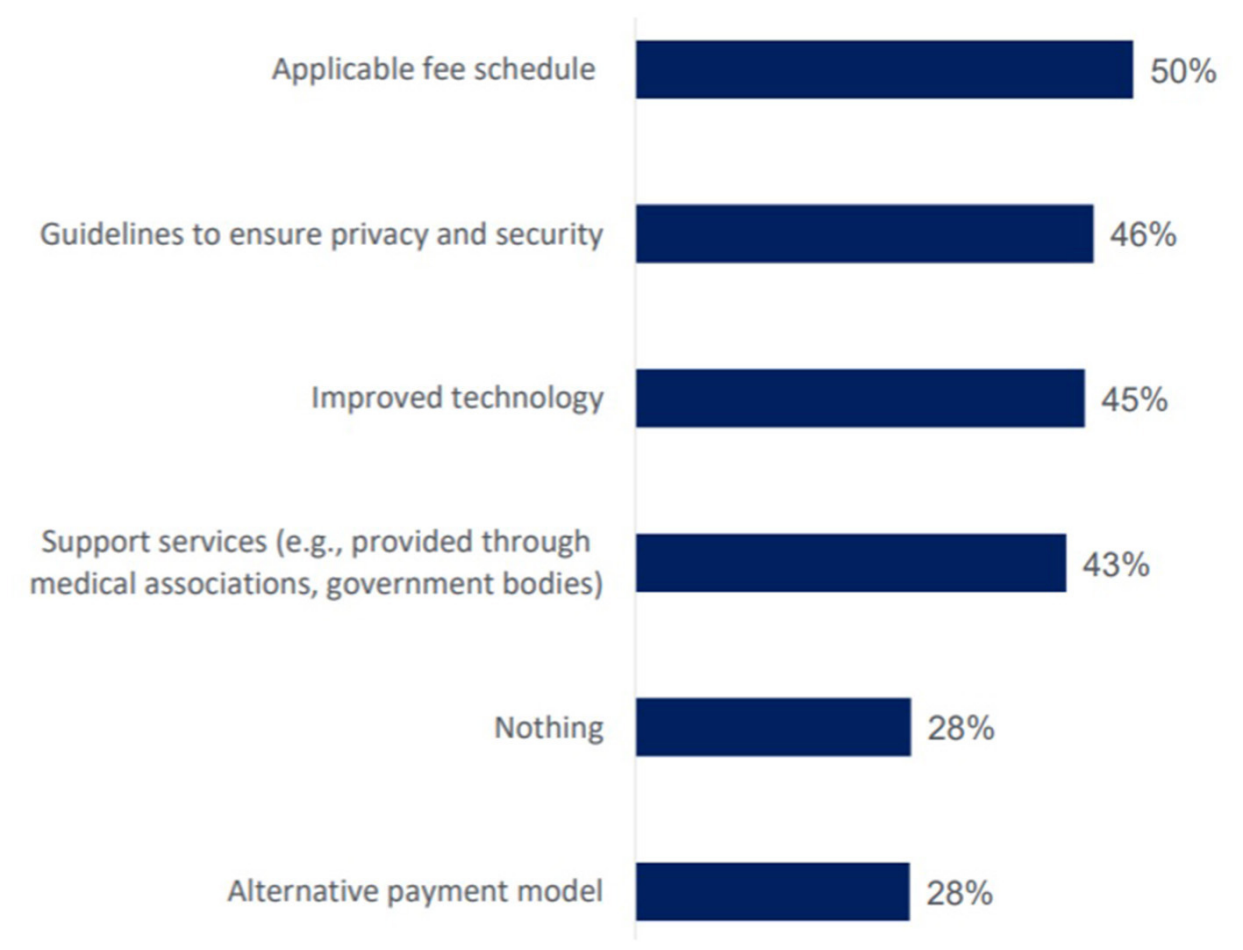
Factors Canadian physicians report would support electronic communication with patients in practice. Sourced from a survey of 1,400 Canadian Physicians in 2018 (13).

As noted in Figure 5, the Canadian Physician Survey explored the supports required by physicians to advance virtualization of care. The results show that the actual technology is important, but remuneration (the fee or billing schedule) was the most reported issue. Physicians also need support to make these digital tools safe, secure and effective parts of their practice.

## Empirical Case Examples

### Empirical Case Example—EMR Adoption in Canada

In 2006, Canada lagged peer countries in national reported adoption of EMRs in primary care. The Commonwealth Fund International Survey of Primary Care Physicians found that leaders like the UK, New Zealand and the Netherlands had almost completed the digitization of primary care records. At 23% adoption in 2006, Canada ranked among the lowest of the 11 participating countries (14).

For some, an early business case for EMR adoption was emerging: efficiencies for submitting billings to provincial and territorial ministries of health; enhanced quality of record keeping, particularly with respect to legibility; and improved patient safety relating to prescribing and medication management (including legibility of prescriptions, comprehensive medication profiles and basic clinical decision support tools).

However, to most clinicians, the value proposition of EMRs was neither clear nor apparent. At that time, the body of clinical evidence relating to the benefits of EMRs for clinicians and patients was limited. Local Canadian evidence was even sparser. Between 2010 and 2020, more papers were published in the Canadian context outlining some of the value proposition of EMRs to clinicians, including EMRs' return on investment for clinical practices (15) and the ability to leverage EMRs for population-level health management and insights (16).

The technological infrastructure available to clinical practices in the initial phase of EMR adoption (mid-2000s to 2015) was also a barrier. Not all clinics were using computers, and most did not have reliable high-speed internet access. In addition, most clinicians and health care workers did not have adequate education and training relating to the use of technology in clinical practice.

Furthermore, incentives to adopt digital health tools such as EMRs were non-existent. As noted above, adoption occurs slowly in fee-for-service systems without modernized remuneration to support the implementation and appropriate use of digital health tools.

In the context of this misalignment between the perceived efforts and benefits of implementing new technologies, and the inertia that resulted, a catalyst was required to spur adoption.

Specifically, it had become clear that clinicians, policymakers, vendors and other stakeholders would need to collaborate to address this gap. Leading Canadian provinces and territories, including Quebec, British Columbia, Alberta, Ontario, Nova Scotia and the Northwest Territories, established change management initiatives in the form of EMR Support programs.

These programs typically leveraged a federal and provincial/territorial funding partnership, and critically, involved a jurisdictional medical association as well. Each program was uniquely tailored to the province or territory in question, but most shared a similar template influenced by Infoway's National Change Management Framework (17). In addition, the programs were underpinned by two fundamental elements: financial incentives for clinicians to adopt EMRs, and a Clinician Peer to Peer Network to support clinicians throughout their EMR adoption journey with regards to change readiness, education, training, implementation and optimization of EMRs for improving patient care.

These EMR support programs acted as a critical change management catalyst to advance digital health in primary care. Data noted in Figure 2 demonstrate the success of this catalyst: increasing adoption of EMRs in primary care from 23% in 2006 to 86% in 2019. Crucially, this change initiative played a role in establishing EMR-enabled clinical practice as a modern standard of care (18) and helped to lay the foundation for the rapid pivot to virtual care in 2020.

### Empirical Case Example—Virtual Care During COVID-19

As the EMR case example demonstrated, supporting clinicians through digital health change initiatives is difficult to scale in Canada. Projects have faced challenges when they aim for implementation across organizational or provincial/territorial health system boundaries.

As a result, change initiatives have typically been short term and project-based. The complexity of larger scale, national change is compounded in part by the statutory divisions of health care responsibility within the Canadian federation, and Canada's relatively small, diverse population spread over its vast land mass. Nevertheless, with collaboration between interested policymaking stakeholders (federal, provincial/territorial and clinical), successful change initiatives are possible.

Due to past investments, some of the technological infrastructure required to support virtual care was in place for many clinical environments. By 2015, EHRs were in place in all provinces and territories, with variations in some key types of patient information. While they were an important improvement, this infrastructure was neither interoperable, nor optimized for enhancing the patient or provider experience. Most clinics and clinicians had computers with internet access. EMR adoption in primary care, as previously noted, had improved significantly. However, the ability to access and exchange information between systems—EMRs, Hospital Information Systems (HIS), provincial/territorial EHRs—was still limited, as the Commonwealth Fund's 2019 survey of primary care physicians revealed (19).

**Figure 6:**
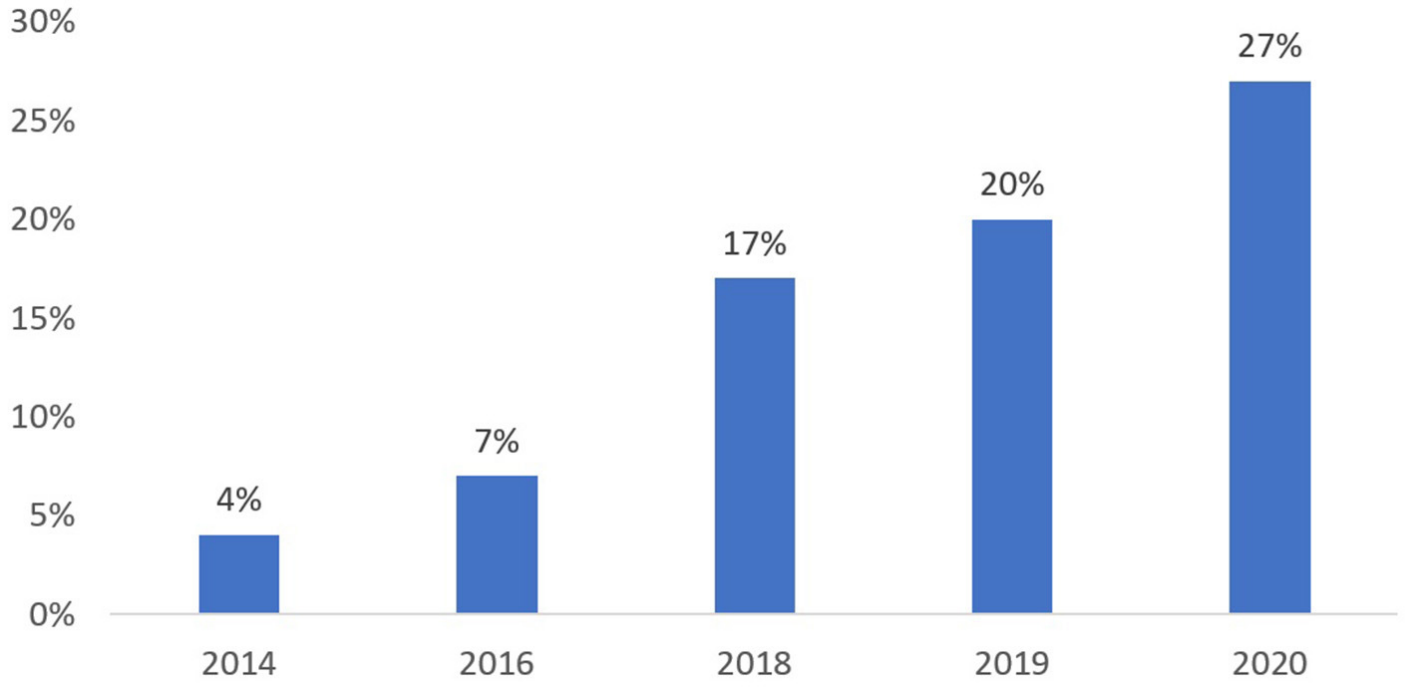
Canadians reporting they have accessed their health record. Sourced from routine representative surveys of the Canadian population conducted between 2014 and 2020, with sample sizes ranging from 1,500 to 6,000 individuals (21).

In the five years between the conclusion of most EMR support programs and the onset of the COVID-19 pandemic (2015 to 2020), numerous initiatives across Canada sought to enhance patients' digital health experiences. Eight in 10 Canadians adults reported they would like access to their own health information, generating momentum through provincial/territorial electronic patient portals, hospital-system associated patient portals, lab service provider results portals, pilot projects relating to virtual care (20) and more. As shown in Figure 6, Canadians were gradually taking advantage of these new services. Eighty six percent of those who access their health information online said they felt more informed about their health, 80% said they can better manage their health and 43% said they avoided an in-person visit (21).

More generally, Infoway survey data indicated a strong interest from patients in engaging electronically with the health system leading up to the pandemic. Electronic prescription renewal, electronic booking and virtual visits were all of interest to a majority of Canadians (21). By 2020, more Canadians were connected on a broader scale, with general access to potent video-capable technology on home computers, tablets or mobile phones.

While the value proposition and benefits of more advanced, patient-focused digital health tools were becoming more evident to patients, they remained unclear to clinicians prior to the pandemic. This scenario was reminiscent of the EMR case example, in which a slowly emerging body of clinical evidence on the value of patient-focused virtual care tools had yet to make an impression on clinicians, who would be the ones required to make investments of time and money to effect their implementation.

A prescient 2018 paper by Shaw et al. (22) about virtual care in the province of Ontario concluded that, “Policy planning for virtual care needs to shift toward a stronger focus on patient engagement to understand patients' needs.”

While Canada had been an early pioneer in telehealth, these services were not available at scale, making up a relatively small proportion of billable visits (23). Some of the conditions were present for virtual care to take place, in the form of video visits, telephone visits and secure messaging, but a key ingredient was missing: an incentive to move away from the status quo of health care delivered almost exclusively in-person. Once again, true digital transformation had not accompanied advancing digitization within the health system. Digital health transformation requires a mixture of the right digital health tools, innovative models of care and appropriate policies, as well as relevant change management mechanisms to support well-designed processes, a virtual-first mindset and strong clinician and patient engagement.

Prior to the pandemic, remuneration for physicians providing virtual care services was noticeably absent in most Canadian provinces and territories. Without modern remuneration models reflecting changing technologies and patient expectations, clinicians had limited incentive to invest in added technological features or workflow modifications, even if these changes would enable efficient, patient-focused virtual care. This reticence was particularly resonant for clinicians in fee-for-service models. Several publications and reports have commented on this proverbial elephant in the room (24–26).

In the absence of credible incentives to innovate, inertia sets in. However, incentives and disruption arrived in the form of the COVID-19 pandemic in March 2020. COVID-19 created an urgency within global health care systems and clinical communities to rapidly pivot toward adopting digital technologies to enable virtual care.

Within the context of the pandemic, the value proposition of virtual care was suddenly very clear to clinicians, patients and health care system policy makers, “…because it provides access to medical care that is timely, convenient, efficient, and safe with reduced risk of transmission (27).” Bhatia et al. neatly summarize the new thinking required by decision-makers to both quantify the value of virtual care in the context of COVID-19, and to redesign care. They suggest thinking about the Costs of Physical Contact (CoPC), “*…a new dimension against which to measure health*,” (28) avoiding physical interactions in health care unless required.

At this critical juncture, several additional catalysts were added to the mix. The first was a coordinated effort by provincial/territorial governments and medical associations to surmount a key policy obstacle for virtual care: the implementation of temporary billing (fee) codes that allowed physicians to be remunerated for providing a visit virtually (by telephone or video) rather than in-person. Numerous clinical organizations also created change management educational materials (videos, how-to guides, webinars) to support clinicians in the rapid pivot toward virtual care. These materials were essential to complement the billing codes. Many of the tools, processes and policies had been built over decades of work providing telehealth to rural and remote communities and building capacity in remote monitoring programs.

The second additional catalyst was a collaboration between federal and provincial/territorial governments to make rapid investments in high yield virtual care tools to facilitate further virtualization of care for patients during COVID-19. These investments accelerated remote patient monitoring (also called home health monitoring), patient portals providing access to COVID-19 and other test results, and virtual care platform licensing agreements. The rapid deployment of these tools, made possible by the EHR, EMR and other infrastructure investments discussed earlier, was essential for tasks like remotely accessing patient information and sharing test results.

**Figure 7:**
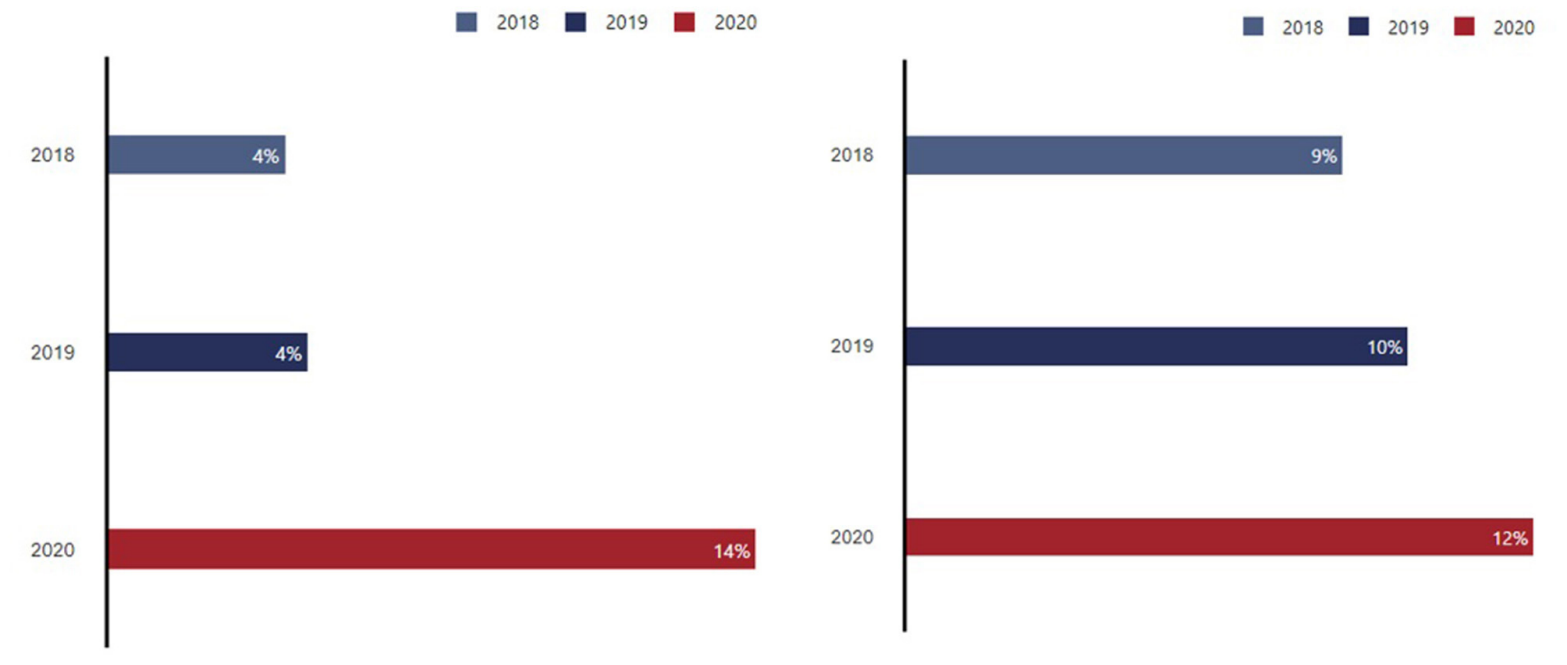
Portion of Canadian adults who have ever had virtual video visit **(left)** or a virtual messaging visit **(right)** 2018–2020. Sourced from routine representative surveys of the Canadian population conducted in 2018, 2019 and 2020, with sample sizes ranging from 2,200 to 6,000 individuals (21).

The results of these and other drivers was an increased use of virtual care. As shown in Figure 7, in August 2020 the Canadian Digital Health Survey captured increases in reported use of virtual visits. While virtual care use has fluctuated throughout the pandemic, it remains significantly more prevalent than before.

**Figure 8:**
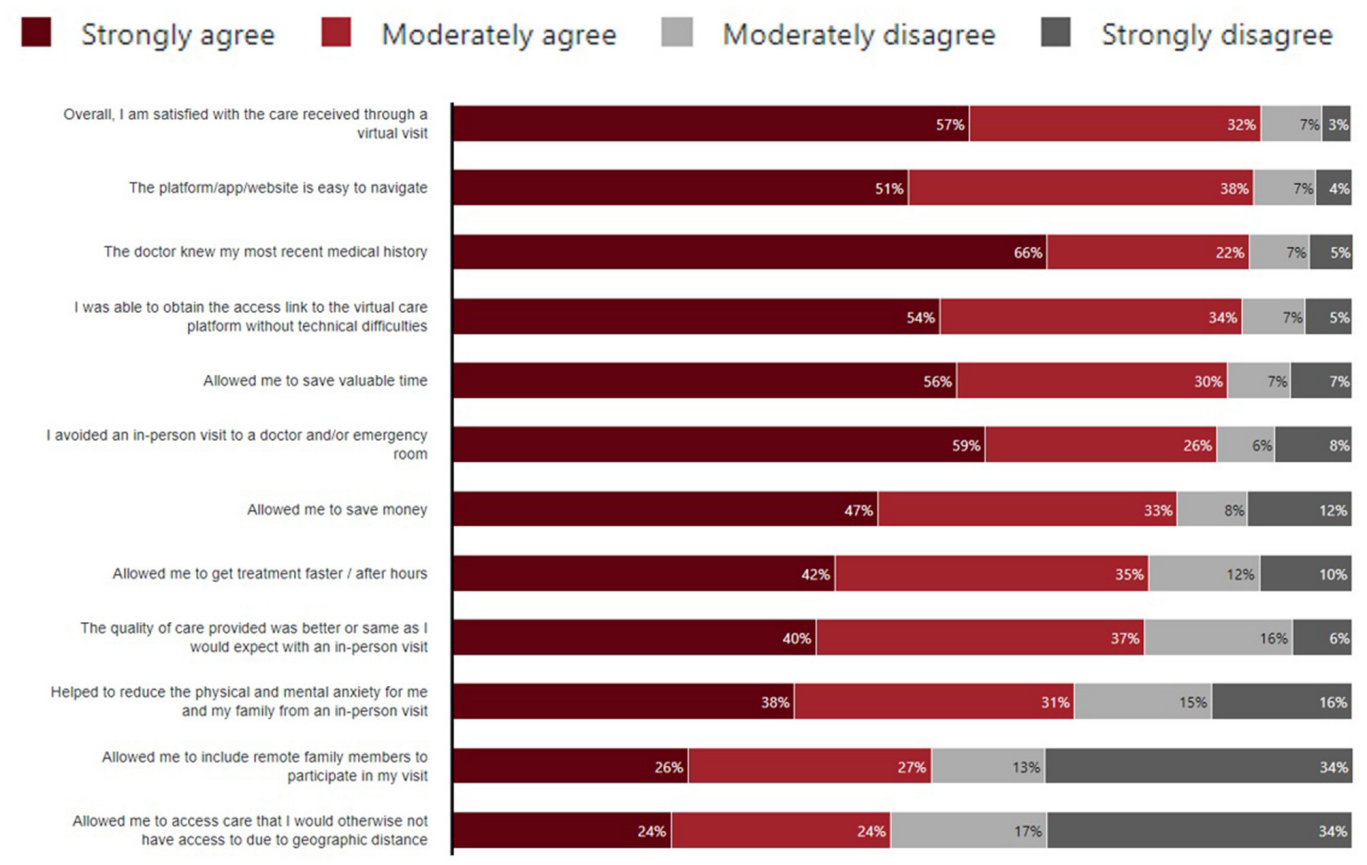
Canadians' experience with their most recent virtual visit, had they experienced one, 2020. Sourced from a routine representative survey of the Canadian population conducted in 2020, with sample sizes of 6,000 individuals (21).

Early data from patient experiences with virtual visits, noted in Figure 8, has shown promising value for Canadians and the health system, thus creating momentum to sustain and optimize virtual modes of care delivery.

This case example demonstrates that a public health crisis of enormous proportions was the initial catalyst required to move Canada's health system from a state of inertia regarding virtual care into a more dynamic state that benefits patients, clinicians and the health system.

Part of the challenge has been business cases that focused on the outcomes for health system funders and clinicians. While evidence is mounting that virtual care can offer significant benefits from both those perspectives, the complex trade-offs, uncertainties and upfront investment are sufficient to dampen progress. With the benefit of new evidence around patient impacts and the significant value in areas like time and financial savings, the overall value proposition becomes much stronger when all perspectives are considered.

In effect, the crisis produced a normalization of virtual care in just a few months, significantly condensing a process of transformation that transpired over years in sectors like travel and banking.

## Discussion

Implementing and optimizing new technologies in Canada's health system has been a challenge for over two decades. This paper has shown that one key element of this challenge has been the mismatch between the efforts and investments required, and the perceived or realized value for stakeholders. As these two case examples have outlined, this misalignment can be overcome with coordinated change management efforts involving the collaboration of multiple stakeholder groups, such as clinician groups, governments and citizens/patients.

Admittedly, the rapid adoption of virtual care has been imperfect. Some clinicians have described it as “making it up as we go along.” (29).

While this may be hyperbole, the transition to a virtual-first mindset in the Canadian health care landscape will require longer-term consideration in order to create sustainable, meaningful change that leads to high quality, safe virtual care in the future.

Clinicians and patients need to know when the use of virtual care is most appropriate and will have the greatest benefit for them. Clinicians and patients will benefit from the refinement of clinical workflows and access to better, more standardized, truly interoperable virtual care tools. Clinicians and the broader health care workforce need additional support and training to supplement what educational institutions have not yet included in their curricula. Patient and caregiver advocacy groups must continue to play a key role in enhancing broad digital health literacy, and in encouraging governments to maintain the momentum around high-quality, patient-centered virtual care that meets the goals of the Quadruple Aim.

Finally, governments and clinician groups will need to collaborate on continuous efforts to modernize remuneration structures to incentivize the provision of modern health care.

Digitization alone is not transformation (30). When the conditions for digital health adoption are present, further catalysts and change management efforts are needed to alleviate the misalignment between perceived costs and benefits to health stakeholders, and to free the modern health system from the inertia of a past status quo. To achieve a goal of full digital transformation, we must invest in change.

## Author Contributions

This article has been primarily authored by RB, with contributions by SH, KB, and JK. All authors agree to be accountable for the content of the work.

## Funding

Funding for this paper has been provided by Canada Health Infoway, a Not-for-Profit organization funded by Health Canada.

## Conflict of Interest

The authors declare that the research was conducted in the absence of any commercial or financial relationships that could be construed as a potential conflict of interest.

## Publisher's Note

All claims expressed in this article are solely those of the authors and do not necessarily represent those of their affiliated organizations, or those of the publisher, the editors and the reviewers. Any product that may be evaluated in this article, or claim that may be made by its manufacturer, is not guaranteed or endorsed by the publisher.
